# Global validation of the Coronavirus Anxiety Scale (CAS)

**DOI:** 10.1007/s12144-021-02583-w

**Published:** 2021-12-02

**Authors:** Theo Lieven

**Affiliations:** Institute for Mobility, University of St. Gallen, Bahnhofstr. 8, 9000 St. Gallen, Switzerland

**Keywords:** Coronavirus Anxiety Scale, cross-cultural, model invariance, multigroup analysis

## Abstract

The five-item Coronavirus Anxiety Scale (CAS) was found to be a useful and valid mental health screener. Participants in the respective surveys were mostly from single countries such as the US, Turkey, Mexico, or Brazil. However, a cross-cultural re-examination is lacking. This study fills this gap. In several multigroup confirmatory factor analyses with 25 countries from five continents as groups, sex and age as groups, and different stages of concern with COVID-19 infection, CAS was found to be invariant across all groups; this indicates that CAS is appropriate for meaningfully comparing the results across different groups. On a global basis, Coronavirus anxiety did not differ between female and male participants. Regarding age, however, younger individuals suffered more from anxiety of the pandemic. Individualistic cultures and those with low power distance such as in the Western hemisphere had higher COVID-19 anxiety. CAS values were also higher for those individuals who had been infected by COVID-19, those whose relatives had been infected, and those who experienced COVID-19-related death in the family. Overall, CAS is a parsimonious, valid, and reliable mental health screener on a global basis.

## Introduction

Since the outbreak of the COVID-19 pandemic, millions of individuals have been infected. Many of them had to be quarantined and isolated from their family and friends. Some had to be hospitalized in COVID-19 care units. Roughly two in a hundred died (CSSEGIS n.d.). At the beginning, mostly elder citizens and those with preexisting diseases seemed to be affected; however, insights emerged that anyone could be infected. Even if the disease was mild, “long Covid” could be frustrating; long Covid refers to signs and symptoms that develop during or after an infection consistent with COVID-19, continue for >12 weeks, and are not explained by an alternative diagnosis (NICE guideline [NG188], 2020). Effective drugs do not yet exist for COVID-19, and breakthrough infections have been noted in some people receiving vaccines. In this environment of uncertainty and doubt, fear and anxiety thrive (Gu et al., 2020). Between January 1, 2020, and January 29, 2021, anxiety disorders increased by 25.6% worldwide (COVID-19 Mental Disorders Collaborators, 2021).

In the early stage of the COVID-19 outbreak, on January 26, 2020, the National Health Commission of China published “A notice on the issuance of guidelines for emergency psychological crisis intervention in pneumonia for novel coronavirus infections” (National Health Commission of China, 2020). The Chinese government intended to mitigate the consequences of psychological and mental diseases caused by the epidemic, such as loneliness, anger, fear, or anxiety, which could lead to attack, self-harm, or even suicide (Moitra et al., 2021), likely drawing from studies on the 2003 SARS outbreak, when fear, boredom, loneliness, and anger were observed (Maunder et al., 2003). Lee and Crunk (2020) reported that fear plays a substantial role in mental disorders caused by COVID-19; the risk of anxiety (e.g., COVID-19 anxiety) increases by more than 20% in the presence of neuroticism (John & Srivastava, 1999).

After the worldwide spread of COVID-19, psychologists, psychiatrists, and other mental health workers recommended timely mental health care, particularly for psychologically vulnerable patients (Xiang et al., 2020). Most health care starts with a diagnosis in which it is determined whether and to what extent an individual is affected by a disease. This determination often uses scales derived from constructs composed of several items that are related to a specific disorder. Until autumn 2021, more than 150 research papers were published regarding COVID-19 scales; most of them used the Fear of COVID-19 Scale (FCV-19S), which was developed by Ahorsu et al. (2020). FCV-19S has been verified in many countries (PubMed n.d.). Results are inconclusive as to how many factors FCS-19S should have. Caycho-Rodríguez et al. (2021) evaluated the model in seven Latin American countries and concluded that the two-factor model was the most reasonable. They were the first to analyze the cross-cultural validity of the FCV-19S by the existence of measurement invariance across countries. They could not find overall scalar invariance, which does not guarantee that the intercepts of the structural model are equal across all seven countries. Without invariance, however, comparisons between the countries could lead to doubtful results because the models were understood differently by respondents in the different cultures.

Another cross-cultural analysis of the FCV-19S was executed by Lin et al. (2021) in 11 countries (Bangladesh, UK, Brazil, Taiwan, Italy, New Zealand, Iran, Cuba, Pakistan, Japan, and France). They noted the one-dimensional factor solution to be the most suitable. Measurement invariance, however, could not be found for all countries.

Another scale—the five-item Coronavirus Anxiety Scale (CAS)—was presented in a timely manner after the outbreak of the pandemic (Lee, 2020a)^1^. This more parsimonious scale was validated several times by Lee et al. (2020b) and Lee (2020a) and found to be a useful mental health screener. Lee (2020b) confirmed the importance of the CAS by demonstrating a significant increase in the explained variance in depression, generalized anxiety, and anxiety when the CAS was added to sociodemographic factors, COVID-19 factors (positively tested, knowledge about infections), and vulnerability factors (neuroticism, health anxiety, reassurance-seeking).

Burkova et al. (2021) analyzed anxiety due to COVID-19 in 23 countries by using the Generalized Anxiety Disorder Scale (GAD-7) and the State Anxiety Inventory. They reported scores on both scales to be higher in individualistic countries and those with a low power distance, which refers mostly to the Western hemisphere. However, Burkova et al. (2021) did not test the scales for measurement invariance across cultures, precluding cross-country comparisons.

Because of its parsimonious structure, the CAS can be an excellent choice when determining disorders caused by COVID-19 and may serve as a standard worldwide. However, as Lee et al. (2020b) indicated, the CAS should be validated in countries outside the US, in English or its translated versions whenever necessary, as has been performed with the FCV-19S. The CAS has been validated in Turkey (Evren et al., 2020), Pakistan (Ashraf et al., 2020), Mexico (García-Reyna et al., 2021), and Brazil (Padovan-Neto et al., 2021), with high internal consistency (Cronbach’s α > .82) and sufficiently high fit indices in confirmatory factor analyses (CFAs). However, to determine the worldwide applicability of the CAS with internationally comparable results, a multigroup analysis on measurement invariance across many countries is necessary, as has been performed for the FCV-19S (Caycho-Rodríguez et al., 2021; Lin et al., 2021). To test the CAS for measurement invariance across different groups is the contribution of this study. The rest of the paper is distributed as follows. The choice of countries and participants as well as the applied method will be described. Results, included regional differences, are reported. A final discussion concludes the article.

## Methods

### Design

The CAS was administered in online surveys in 25 countries. The countries were chosen as a convenient sample to cover all six continents, adding up to most world population (more than 50%) and to represent most global cultures (Australia, Austria, Belgium, Brazil, Canada, China, Denmark, Finland, France, Germany, India, Italy, Japan, Netherlands, Norway, Portugal, Russia, South Africa, South Korea, Spain, Sweden, Switzerland, Taiwan, the UK, and the US).

### Participants

The participants were recruited by an international provider for data analyses and market research with several subsidiaries worldwide. They mostly have their own panels of potential survey participants (only in South Africa, the participants were recruited by an outside provider). The sample size was approximately 400 participants per country. Because statistical inferences are sensitive to sample size, the same number of cases in each country was chosen. To get a sample demographically as close as possible to the countries’ population, a first set of some dozen potential respondents was invited by email. Those who answered were evaluated on their fit to the overall demographics. The next set was then invited to complement the first set regarding the match with sex or age (as an example, in case the country’s distribution of sex is 50% female vs. 50% male and the first set of responses had 30% females and 70% males, the second set tried to compensate for this by including an extra number of female participants). Although this procedure is rather heuristic and not representative, it resulted in acceptable allocations in the assessed samples with only some major deviations from actual distributions (in Japan, Russia, and the US, females were underrepresented by approximately 15%). In the worldwide data, however, female participants accounted for up to 46.4%, whereas the total distribution is 49.3%. The average age of the samples in online surveys typically tends to be younger than the actual ages in any country. However, the demographic structure of the assessed data seemed to be sufficiently appropriate for the current research question.

### Measure

The five items of the CAS (Lee, 2020a) were presented to the participants: dizziness, sleep disturbances, tonic immobility, appetite loss, and abdominal distress. Participants had to rate them on the same 5-point scale as in the original version (How often have you experienced any of the following in the past 2 weeks? 0 = not at all; 1 = rare, less than a day or two; 2 = several days; 3 = more than 7 days; 4 = nearly every day over the last 2 weeks). In addition to age and sex, three more variables were assessed: (1) the participant’s own history of COVID-19 infection; (2) a relative’s history of COVID-19 infection; and (3) a relative’s death from COVID-19.^2^ The original CAS version in English was administered in the US, Australia, Canada, India, South Africa, and the UK. For the other countries, the translations of the questionnaires were conducted by a professional translation agency, which employs graduate translators who are native speakers of a particular language. The original English AS scale was translated into 14 languages: German (for Germany, Austria, and Switzerland), French (for France, Belgium, Canada, and Switzerland), Portuguese (for Portugal and Brazil), Chinese (for China and Taiwan), Danish (for Denmark), Finnish (for Finland), Italian (for Italy), Japanese (for Japan), Dutch (for The Netherlands and Belgium), Norwegian (for Norway), Russian (for Russia), Korean (for Korea), Spanish (for Spain), and Swedish (for Sweden). These 14 versions were translated back into English to assure that the correct meaning of the questions. Some other translations already exist on “The Coronavirus Anxiety Project” (n.d.), which were compared with the own versions. Only minor differences were noted, with no shifts in meaning. This may be due to the easily comprehensible everyday topics of the respective questions. Data collection was conducted during March 18–31, 2021.

## Results

### Descriptive Results

In total, 10,232 respondents participated, with an average of approximately 400 per country. The descriptive details are depicted in Table 1. The average CAS scores range in the middle of what had been reported in the past. In some studies, they were higher at 6.66 (Evren et al., 2020) or 8.62 (Lee, 2020a) and in some, they were lower at 2.15 (García-Reyna et al., 2021) or 2.66 (Padovan-Neto et al., 2021).

**Table 1:** Descriptive data per country

Country	N	Female	*M*_Age_	SD_Age_	Dizziness	Sleep Disturbances	Tonic Immobility	Appetite Loss	Abdominal Distress	Coronavirus Anxiety Scale ^a^	Incidence until March 31, 2021^b^	Was infected himself with COVID-19	A relative was infected with COVID-19	A relative died of COVID-19
Australia	400	48.9%	46.5	12.3	.48	.57	.51	.49	.49	2.53	115	10	47	18
Austria	407	43.7%	40.6	12.2	.68	.85	.80	.65	.73	3.71	6,166	52	131	19
Belgium	406	48.9%	41.1	13.8	.55	.94	.67	.62	.73	3.51	7,614	51	130	27
Brazil	413	49.9%	34.6	9.5	.58	1.17	1.02	.84	.78	4.40	5,998	73	238	59
Canada	422	53.9%	48.3	11.5	.46	.68	.49	.41	.44	2.47	2,619	12	69	20
China	422	46.3%	35.4	8.1	.66	.77	.65	.68	.59	3.36	6	2	13	5
Denmark	411	46.5%	45.2	14.3	.58	.74	.65	.61	.70	3.28	3,993	28	80	10
Finland	405	43.2%	37.6	12.7	.61	.73	.64	.60	.59	3.16	1,398	12	52	14
France	417	49.4%	43.9	12.1	.49	.93	.63	.55	.69	3.29	7,208	32	132	20
Germany	416	48.2%	44.1	13.5	.64	.79	.83	.63	.71	3.59	3,394	14	85	23
India	420	38.3%	32.3	8.2	1.60	1.51	1.30	1.42	1.21	7.03	885	61	153	62
Italy	416	51.2%	41.6	11.8	.62	.99	.59	.62	.68	3.50	5,929	31	137	27
Japan	399	34.8%	45.0	11.7	.37	.43	.25	.37	.62	2.03	375	8	12	2
Netherlands	402	48.2%	41.9	13.3	.66	.82	.70	.76	.74	3.68	7,545	35	122	22
Norway	408	54.4%	38.0	13.8	.70	.83	.76	.75	.77	3.80	1,772	34	87	22
Portugal	407	47.2%	38.3	11.7	.22	.77	.51	.41	.34	2.25	8,059	23	142	26
Russia	412	35.5%	37.1	10.1	.30	.36	.40	.25	.20	1.51	3,079	87	144	18
South Africa	411	57.8%	33.7	9.8	.61	.98	.76	.57	.63	3.54	2,610	28	161	86
South Korea	415	42.4%	42.5	10.2	.41	.47	.42	.51	.40	2.22	202	4	15	14
Spain	414	45.4%	41.5	11.2	.68	1.08	.84	.60	.56	3.75	7,024	32	154	28
Sweden	414	47.0%	39.4	12.5	.82	.90	.88	.86	.92	4.39	7,970	69	179	34
Switzerland	403	53.5%	41.8	13.0	.55	.81	.76	.61	.81	3.53	6,946	51	132	26
Taiwan	404	44.5%	38.5	9.8	.58	.61	.66	.57	.52	2.95	4	5	12	8
UK	394	49.5%	44.6	12.1	.71	.93	.70	.71	.72	3.78	6,422	39	104	29
USA	394	31.8%	47.1	14.7	.89	.95	.78	.81	.84	4.27	9,203	57	119	32
Total	10,232	46.4%	40.8	12.6	.62	.83	.69	.64	.66	3.42		850	2,650	651

^a^Sum of the scores of the five Coronavirus Anxiety Scale items.

^b^Total COVID-19 infections from January 2020 to the end of March 2021 per 100,000 population (CSSEGIS n.d.).

The appropriateness of comparisons across groups (countries, sex, age, etc.) depends on the invariance of the measurements, which guarantees that in all groups, the same parameters are measured using the same instruments. Since in CFA, the fit of the data to the measurement function can be assessed, it can be determined whether and how this fit aggravates when one or more other groups of data are added in the model. Thus, the data were first examined on their suitability to analyze them in exploratory factor analysis (EFA) and subsequently tested regarding invariance across countries, sex, age, and the groups that were influenced by the COVID-19 virus.

### Exploratory Factor Analysis (EFA)

In the aggregated global sample, the reliability index was similarly high, as in Lee et al.'s (Lee, 2020c) analysis (Cronbach’s α = 0.918). The Kaiser–Meyer–Olkin test (= .895, *p* < .001) and Bartlett test indicated sufficient support for a factorable solution. Principal component analysis resulted in one single factor explaining 75% of the total variance. Factor loadings were ≥0.84. These figures were similar for nearly all countries, with all Cronbach’s α values > .9 and the average extracted variance (AVE) of the data > .720. In four countries (Brazil, India, Portugal, South Africa), all Cronbach’s α values were < .890 and the AVE was < .690; nevertheless, the results remain reasonable for a factor solution.

### Confirmatory Factor Analyses (CFA)

A multigroup CFA with the 25 countries as groups was conducted. To assess comparability across countries, data were tested on invariances across the groups (Steenkamp & Baumgartner, 1998) using four models:

A An unconstrained model to assess configural invariance (or pattern invariance) to assess for the fit of the same one-factor structure with five items in all countries.

B A model with measurement weights (factor loadings) constrained to be equal across groups (metric or “weak” invariance) to assess that not only the same items load on the same factors for all groups but also the actual magnitudes of the factor loadings are the same across groups for each respective item. In case of metric invariance, different scores on the items can be meaningfully compared across groups.

C A model with factor covariances constrained to be equal; because in this CFA only one factor and no structural model exists, only the variance of the one-factor CAS will be constrained to be equal.

D A model where all variances of the residuals (errors) were constrained to be equal across all countries.

In case all items are metrically invariant, and the factor variance and the error variances are invariant cross-nationally as well, the model is equally reliable across countries (Steenkamp & Baumgartner, 1998).

The analyses were conducted with the AMOS analytic tool for structural equation modeling (Arbuckle, 2014), using the maximum likelihood estimator^3^. Three criteria had to be met to support the invariance hypotheses (Cheung & Rensvold, 2000). The decrease of the Tucker–Lewis index (TLI) from one model to the next had to be low (|ΔTLI| ≤ .05), and the difference of root-mean-square error of approximation (RMSEA) and the probability of close fit had to be nonsignificant (*p*_close_ ≥ .05). For a description of the fit indices and cutoff values, see the note below Table 2.

**Table 2:** Fit Indices and Factor Loads per Country

	CFI	TLI	RMSEA	*p*_close_	SRMR	Dizziness	Sleep Disturbances	Tonic Immobility	Appetite Loss	Abdominal Distress	AVE	Cronbach’s α
Australia	.999	.998	.026	.679	.0065	.83	.82	.94	.88	.87	76%	.942
Austria	.994	.987	.076	.119	.0122	.86	.86	.90	.89	.87	77%	.943
Belgium	.993	.982	.076	.141	.0167	.79	.75	.90	.83	.74	65%	.901
Brazil	.991	.978	.074	.156	.0191	.68	.74	.79	.78	.87	60%	.875
Canada	.996	.991	.061	.287	.0124	.87	.74	.87	.91	.89	74%	.927
China	1.000	1.000	.011	.725	.0075	.74	.72	.76	.86	.90	64%	.906
Denmark	.996	.991	.056	.347	.0122	.75	.84	.86	.85	.87	70%	.915
Finland	.992	.983	.076	.118	.0152	.72	.80	.86	.88	.87	69%	.915
France	.998	.995	.041	.559	.0127	.81	.74	.88	.88	.84	69%	.915
Germany	.999	.998	.027	.637	.0091	.72	.77	.78	.88	.92	67%	.915
India	.966	.993	.043	.531	.0149	.74	.72	.82	.78	.84	61%	.886
Italy	.996	.988	.065	.259	.0118	.81	.75	.89	.79	.84	67%	.916
Japan	.991	.976	.087	.068	.0189	.78	.83	.80	.92	.76	67%	.903
Netherlands	.996	.990	.067	.219	.0100	.89	.81	.87	.90	.89	76%	.941
Norway	.993	.983	.080	.107	.0142	.82	.81	.89	.81	.85	70%	.924
Portugal	.996	.990	.048	.456	.0169	.70	.70	.75	.81	.84	58%	.856
Russia	.995	.987	.066	.233	.0151	.73	.86	.85	.88	.75	67%	.905
South Africa	.999	.997	.022	.671	.0107	.69	.68	.71	.70	.68	48%	.836
South Korea	.997	.994	.051	.415	.0101	.85	.78	.89	.90	.89	75%	.931
Spain	.998	.995	.042	.524	.0098	.83	.75	.91	.79	.79	67%	.910
Sweden	.997	.991	.057	.336	.0103	.72	.80	.87	.87	.88	69%	.923
Switzerland	.999	.999	.022	.716	.0082	.82	.78	.85	.87	.83	69%	.920
Taiwan	.998	.996	.041	.533	.0063	.89	.90	.84	.86	.85	75%	.941
UK	.996	.991	.060	.295	.0118	.88	.81	.88	.88	.88	75%	.935
USA	.997	.989	.071	.204	.0081	.87	.85	.89	.90	.88	77%	.946

Note: CFI = comparative fit index (should be ≥ .95); TLI = Tucker–Lewis index (≥ .95); RMSEA = root-mean-square error of approximation (should ≤ .06); *p*_close_ ≥ .05 (nonsignificant); SRMR = standardized root-mean-square residual (should be ≤ .08), For cutoff values see (Hu & Bentler, 1999). AVE = average variance extracted (should be ≥ 50%, Fornell & Larcker, 1981). Cronbach’s a = reliability index (should be .70 – .90, Tavakol & Dennick, 2011).

**Fig. 1:**
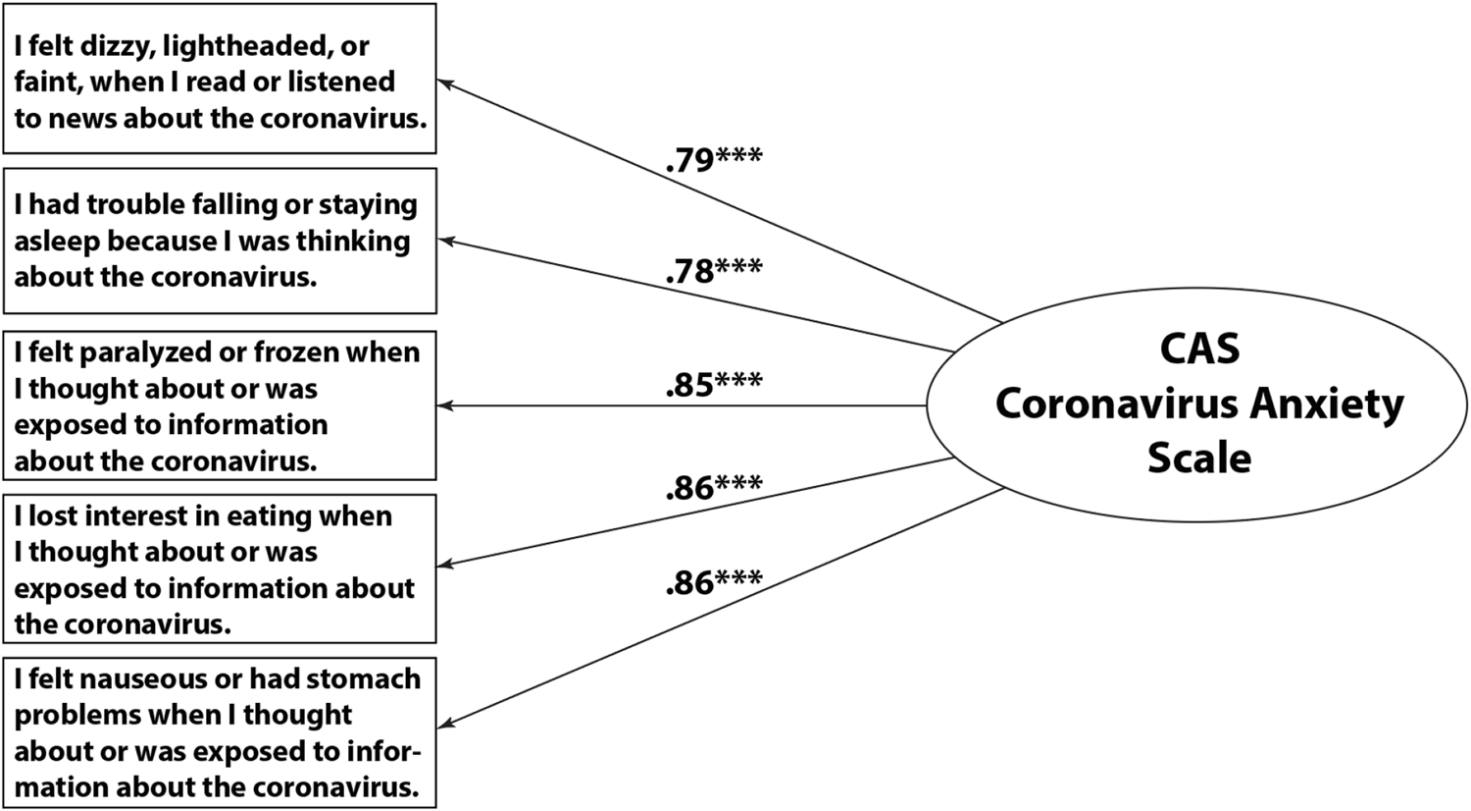
The Coronavirus Anxiety Scale in 25 countries

The unconstrained model A had a good fit (TLI = .963, RMSEA = .0228, *p*_close_ = 1.000; Hu & Bentler, 1999). Model B for metric invariance had an even higher TLI (.971), lower RMSEA (.020), and insignificant *p*_close_ (1.000). Thus, metric invariance was granted. Model C had TLI = .963, an RMSEA = .023, and *p*_close_ = 1.000. Thus, the variance of the CAS factor can be assumed to be invariant across countries. Finally, for Model D, TLI = .925, RMSEA = .033, and *p*_close_ = 1.000. Thus, the difference in TLI in the last step from model C to model D is lower than the threshold of .05 (|ΔTLI| = .038; ΔRMSEA = .010; *p*_close_ = 1.000, not significant). Thus, the model is supposed to be similarly reliable across all countries with the parameters presented in Figure 1. AVE extracted regarding convergent validity was sufficiently high for all items (AVE > 50%), thus meeting the Fornell–Larcker criterion (Fornell & Larcker, 1981).

A similar procedure for the age groups resulted in a slightly worse fit for the unconstrained model with an RMSEA of .062 and a significant *p*_close_ < .001, but still sufficient with a TLI = .978. Steps to models B, C, and D even increased the fit to TLI = .985 and RMSEA = .051, *p*_close_ = .347, not significant. For the sex groups, model A had a TLI = .973, which decreased to .942 in model D; thus, ΔTLI = .031, which is below the threshold. The *p*_close_ values for models A, B, and C were nonsignificant (1.000); however, model D had *p*_close_ < .001 but had RSMEA = .053, thus indicating a good fit. The same held for the groups where the individual was infected/not infected, the groups where a relative was not infected/not infected, and the groups where a relative had died/not died. In all group comparisons, factor loadings, factor variances, and error variances were the same (except some rounding effects): CAS → dizziness = .79, CAS → sleep disturbance = .78, CAS → sonic immobility = .85, CAS → appetite loss = .86, CAS → abdominal distress = .86; this added up to an AVE of 68.6%, which is higher than the required 50% (Fornell & Larcker, 1981). Thus, the CAS (Figure 1) was determined to be completely reliable across all 25 countries, sex, age, and infection experiences, and the respective CAS scores can be appropriately compared across the above groups. Detailed information about fit indices and factor loading for each country can be found in Table 2.

### Cross-Group Comparisons

#### CAS Depending on Sex and Age

In total, CAS did not significantly differ regarding sex (male: 3.41, female: 3.43). This applied for 21 out of the 25 countries. CAS differed significantly between females and males in the US (females 3.05, males 4.90, *p* < .01), in Switzerland (females 4.11, males 2.85, *p* < .01), in Russia (females 2.01, males 1.13, *p* < .05), and in Germany (females 4.24, males 2.98, *p* < .01).

Elder participants had a lower CAS (*r* = –.200, *p* < .001). This applied significantly to 17 out of the 25 countries, see Table 3. This is consistent with the findings of Kowal et al. (2020) who found that higher levels of stress due to the pandemic were associated with younger age.

**Table 3:** CAS and age groups per country

	20 - 29 years	30 - 39 years	40 - 49 years	50 - 59 years	60 - 69 years	Sig.
Australia	4.74	3.64	2.69	1.36	1.22	***
Austria	4.15	6.58	2.24	1.83	1.14	***
Belgium	4.25	4.60	3.08	2.41	1.51	***
Brazil	4.73	4.54	3.72	4.09	3.57	n.s.
Canada	3.63	3.04	2.63	1.95	1.77	**
China	3.95	3.46	2.72	1.14	N/A	**
Denmark	7.00	4.85	2.70	1.29	0.99	***
Finland	4.38	4.18	2.36	1.14	1.61	***
France	5.38	3.37	3.22	2.49	2.59	**
Germany	4.89	4.62	3.71	2.40	2.26	***
India	6.83	7.56	5.92	7.80	4.00	n.s.
Italy	3.39	3.98	3.57	3.04	2.86	n.s.
Japan	4.28	2.22	2.16	1.17	0.78	***
Netherlands	5.80	4.92	2.58	1.88	1.68	***
Norway	4.44	5.02	3.54	2.31	0.93	***
Portugal	2.54	2.59	2.25	1.09	1.53	**
Russia	1.64	1.49	1.38	2.08	1.75	n.s.
South Africa	4.12	3.05	2.68	4.61	3.00	n.s.
South Korea	2.82	2.54	2.68	1.96	1.73	n.s.
Spain	5.10	4.53	3.13	2.77	2.37	***
Sweden	4.58	5.29	4.47	3.50	2.73	n.s.
Switzerland	3.75	5.18	3.40	2.24	2.09	***
Taiwan	3.09	3.07	2.77	2.71	1.75	n.s.
UK	5.61	5.54	3.31	2.36	1.62	***
USA	6.60	6.65	6.00	2.90	1.19	***
All	4.46	4.24	2.98	2.18	1.65	***

Note: Because of low case counts within the countries, participants below 20 and above 69 years have not been considered.

#### CAS Depending on Concern of COVID-19

Participants who had been infected by COVID-19 (n = 850) had a significantly higher CAS of 5.66 than those who were not infected (CAS = 3.22, t = 14.803, *p* < .001). Participants whose relatives had been infected by COVID-19 (n = 2650) had a CAS of 4.40 compared with those who had not been infected (CAS = 3.08, t = 12.622, *p* < .001). Participants who had a death in their family because of COVID-19 (n = 651) had a higher CAS of 5.5 than those who had no COVID-19-related death in their family (CAS = 3.28, t = 11.852, *p* < .001). Stronger anxiety can also be seen from the correlations between the CAS and the three infection stages: individual infected (*r* = .408, *p* < .05), relative infected (*r* = .468, *p* < .05), and relative died (*r* = .577, *p* < .01).

#### CAS Compared with Incidence Rates Per Country

The variables in Sections *3.4.1* and *3.4.2* were individually assessed per respondent (age, sex, and concern of COVID-19). Several external country-specific key figures were included in the analyses. One of these numbers is the incidence rate per country during the COVID-19 crisis until the end of March 2021 (the month of the execution of this survey). Contrasting CAS with the incidence rate in a one-way analysis of variance resulted in a significant *F*(24,10207) = 22.389, *p* < .001. Tukey’s-b post hoc test revealed 10 homogeneous subgroups (Table 4).

**Table 4:** CAS scores and Incidences

Group	CAS	Incidence
1	2.17	2,408
2	2.41	1,896
3	2.59	2,066
4	2.85	3,342
5	3.19	3,583
6	3.30	3,565
7	3.50	4,535
8	3.58	5,149
9	3.71	5,613
10	7.03	885

Group 10 comprises only India. Its CAS value (7.03) was the highest of all, but the incidence was quite low until that time. India, thus, seems to be an outlier. The reason for the high CAS score will be analyzed in the discussion section. Removing India (Group 10) from the homogeneous subgroups resulted in a significant positive correlation (.931, *p* < .001), thus supporting the plausibility that countries that are strongly affected by COVID-19 display stronger Coronavirus anxiety.

#### CAS Compared with Hofstede’s Cultural Indices

Similar one-way ANOVAs were conducted with Hofstede’s cultural indices (Hofstede, 1980; Hofstede et al., 2010). The outlier India was again excluded from these analyses. Regarding the power distance index (PDI, low = low power distance), eight subgroups emerged with a negative correlation (*r* = –.855, *p* < .01); this implied that countries with a high power distance had a lower Coronavirus anxiety. The same analysis for Individualism vs. Collectivism index (IDV, low = collectivistic, high = individualistic) resulted in seven subgroups with *r* = .982, *p* < .001. Thus, individualistic countries show a higher Coronavirus anxiety than collectivistic countries. Similarly, the indulgence versus restraint index (IVR, low = restraint, high = indulgent) had a high positive correlation with CAS (five subgroups with r = .943, *p* < .05); thus, indulgent cultures had higher Coronavirus anxiety than restraint cultures. Regarding the uncertainty avoidance index (low = low uncertainty avoidance), seven subgroups emerged with *r* = –.893, *p* < .01. Thus, countries with high uncertainty avoidance displayed lower Coronavirus anxiety. The masculinity (MAS) and long-term orientation indices were not significantly correlated with Coronavirus Anxiety Index.

## Discussion

The one-dimensional CAS fit the assessed data well. Fit indices (CFI and TLI) that are well above the cutoff value of .95 are rare. However, this excellent fit is in line with prior research (Evren et al., 2020; Lee, 2020a, 2020b; Lee & Crunk, 2020; Padovan-Neto et al., 2021). The lowest TLI was .971 in Brazil (Padovan-Neto et al., 2021). Thus, the CAS was concluded to be a parsimonious and valid instrument to assess individual anxiety about the Coronavirus.

Although the CAS could be successfully replicated separately in different countries, this study is the first to analyze cross-national validity. The CAS items were shown to be equally reliable across 25 countries across six continents. All invariances—configurational, metric, covariance, and error invariances— were approved, which is also rare. Since such a good fit is so unusual for an international dataset like the one used in this study, one might suspect that the model has unwanted properties, such as redundant items in the one-factor solution. The high reliability indices (Cronbach’s α) could be evidence for such redundancy. Except in Brazil, India, and South Africa, they are well above the suggested level of .90 (Tavakol & Dennick, 2011). However, high reliability indices can be expected from larger scales with many items evaluating the same concept. Next, redundancy is more probable. The item scores (Table 1) correlate significantly, which is a prerequisite to combine them with a factor. However, pairwise t-tests in each country resulted only in a minority of cases assuming that two items had to be treated as equal. Thus, redundancy is not a reason for the good fit of the model. A more likely reason might be the balanced and easy-to-understand questions that make respondents answer consistently, fostering good reliability.

Scores differ significantly across countries. CAS scores ranged from 1.51 to 7.03, consistent with recent research from other countries—6.66 in Turkey, 2.15 in Mexico, and 2.66 in Brazil (Evren et al., 2020; García-Reyna et al., 2021; Padovan-Neto et al., 2021). The reason underlying the high CAS value of 7.03 in India can only be assumed from another recent study (Lieven & Hildebrand, 2016) demonstrating that Indian respondents tended to an extreme response style on the right side of the scale. While the average skewness across all countries was 1.45, it was only .325 in India, indicating that the distribution in India was much less right skewed because of higher ratings. The high CAS score of 8.62 in the inaugural study by Lee et al. (2020a) may be due to the fact that participants were recruited who had experienced significant anxiety, fear, or worry about the disease outbreak. This cohort was therefore likely to have higher Coronavirus-related anxiety than a broader cross-sectional cohort of the representative population. The more it has to be emphasized that the CAS model fits all data well, be it from highly COVID-19 affected persons or less affected respondents.

The differences between the scores across countries may be because of inappropriate translations. However, in India, the same original English questionnaire was used as in the US, but the CAS score was 7.03 and 4.27, respectively. Similarly, the same Portuguese translation was administered in Brazil and Portugal, but scores in Brazil were nearly twice as high as those in Portugal.

The translations of the questionnaire were conducted by professional translators, who were all native speakers in the respective language, and their names and qualifications are known to the authors of this study. A comparison with some available translations on "The Coronavirus Anxiety Project” (n.d.) and a backtranslation of both versions did not show serious differences; only minor ones that did not distort the meaning of the five questions. The questions themselves were comprehensible to most people. Diseases such as dizziness, faint, insomnia, lack of appetite, or nausea are common everyday topics that simply cannot be misunderstood. In any case, why scores differ significantly between countries cannot be analyzed from the perspective of this study. Cultural sensitivities or idiosyncrasies could lead to a different comprehension. These causes cannot be observed in a worldwide view, as was conducted in this study. It could be a promising venue for future research when local researchers take care of these matters.

Cultural comparisons revealed that individualistic and indulgent countries with a low PDI (i.e., Western countries) exhibited stronger COVID-19 anxiety. Asian countries (except India) had a CAS of 2.64; the CAS in the Western hemisphere was 3.51. This is consistent with previous findings from studies in 23 countries (Burkova et al., 2021); however, that study did not verify cross-cultural invariance. Perhaps the cohesion and solidarity with a clear hierarchal society, which is the characteristic of collectivistic cultures with high power distance, helps people overcome anxiety. Surprisingly, uncertainty avoidance did not facilitate anxiety. By contrast, countries with a high uncertainty avoidance index had lower anxiety. It might have been a rational risk aversion instead of psychological uncertainty that had been assessed by Hofstede (1980) and Hofstede et al. (2010). In general, one should approach Hofstede's scale with caution. It is 40 years old, and cultures have changed due to globalization. However, no new cultural scale is available.

Two findings in this study are counterintuitive. One is the stronger anxiety among young individuals. COVID-19 is more dangerous for elder persons, particularly those with preexisting diseases. Younger persons are not yet affected by such diseases and are said to have a stronger immune system. Consequently, one should expect elder individuals to have stronger Coronavirus anxiety. The reason for the unusual finding might be that old people above 70 or 80 were not sufficiently covered by this study. From 20 to 69 years, however, the above finding holds.

We did not use the Rasch DIF model for parsimonious reasons. This seemed to be appropriate because several studies have shown consistent results regarding confirmatory factor analysis and the Rasch DIF (Randall & Engelhard, 2010), particularly for the FCV-19S (Lin et al., 2021).

One limitation of the present study is the unbalanced choice of countries. Fifteen countries were chosen from Europe, and only one each from South America and Africa. However, our results revealed that there is a high probability that any CAS dataset from another country will fit in the existing panel of 25 countries as long as the individual fit indices of that country are sufficiently good.

## Conclusion

Our data indicates that CAS is robust to be disseminated into many other cultures. Compared with the Obsession with COVID-19 scale (OCS, Choi et al., 2020; Lee, 2020c) and the FCV-19S (Ahorsu et al., 2020; Caycho-Rodríguez et al., 2021), the CAS seems to be at least equivalent and has cross-cultural reliability.

## Footnotes

1 The study by Lee et al. (2020a) was published on April 16, 2020. Another article using the term CAS was submitted 3 days later and published on June 2, 2020 (Chandu et al., 2020). The two-factor solution had some shortcomings: Only two items were included for the construct “illness anxiety.” Moreover, only an exploratory factor analysis was conducted, precluding validity assumption. Thus, this study was excluded from the current study.

2 Because these items are personal health questions, we followed the ethical guidance of informed consent (WMA, 2013).

3 Although the CAS items are ordinal data, such type of Likert scales can be taken to be interval scaled (Brown, 2011). Maximum likelihood (ML) estimation in general requires multivariate normal distribution. This assumption is rarely met with empirical data. However, due to its robustness ML estimation exhibits little bias with non-normal data (Benson & Fleishman, 1994).

## Declarations

### Data Deposition

My manuscript has data included as electronic supplementary material.
